# Screening of Proliferation-Related Genes and Pathological Changes in Thiram-Induced Tibial Dyschondroplasia

**DOI:** 10.1155/2022/6209047

**Published:** 2022-07-14

**Authors:** Ali Raza Jahejo, Chen-Liang Zhang, Fazul Nabi, Nazeer Hussain Kalhoro, Qurban Ali Shah, Zohaib Ahmed Bhutto, Jin-Feng Zhao, Jin Yu, Guan-Bao Ning, Ding Zhang, Shu-Ming Chen, Wen-Xia Tian

**Affiliations:** ^1^College of Veterinary Medicine, Shanxi Agricultural University, Jinzhong 030801, China; ^2^Sindh Institute of Animal Health, Karachi, Pakistan; ^3^Faculty of Veterinary and Animal Science, Lasbela University of Agriculture Water and Marine Science, Uthal, Balochistan, Pakistan

## Abstract

**Materials and Methods:**

Three hundred sixty (*n* = 360) broiler chickens were equally divided into control (C) and thiram (T) groups. Furthermore, the C and T groups were dividedinto 8-, 9-, 11-, and 13-day-old chickens.

**Results:**

Clinically, it was observed that broiler chickens of group T had abnormal posture, gait, and lameness, and histopathological results revealed dead and abnormal chondrocytes of T group on day 6. Real-time qPCR results showed that HDAC1, MTA1, H4, and PCNA genes were significantly expressed (*P* < 0.05). HDAC1 was upregulated on days 1, 2, 4, and 6 (*P* < 0.01); MTA1 was upregulated on days 1 and 2 (*P* < 0.01); H4 was upregulated on days 2 and 4 (*P* < 0.01), and PCNA was downregulated on days 1, 2, and 4 (*P* < 0.01). Furthermore, IHC results of HDAC1 protein were significantly (*P* < 0.01) expressed in proliferative zone of day 1 and hypertrophic zone of day 6. MTA1 protein was significantly (*P* < 0.01) expressed on days 1, 2, and 6 in all zones, except prehypertrophic zone of day 2.

**Conclusion:**

In conclusion, the mRNA expressions of HDAC1, MTA1, H4, and PCNA were differentially expressed in the chondrocytes of thiram-induced TD chickens. HDAC1 and MTA1 protein expression found involved and responsible in the abnormal chondrocytes' proliferation of broiler chicken.

## 1. Introduction

Tibial dyschondroplasia (TD) is a disease of broiler chickens manifested by accumulation of rigid mass inside the growth plate (GP), resulted in abnormal posture. Due to TD, more economic loss had been reported worldwide with 40-60% incidence in the flock. Lot of studies had been reported for resolving TD, but its concerned pathogenesis still question mark [1].

Thus, any development regarding TD pathogenicity is of utmost importance. Moreover, researchers have reported that a vascularization and unmineralization in the GP could be the cause of TD [2, 3]. In addition, improper nutrient supply to the chondrocytes stopped the bone development which cause poor calcification of the cartilage matrix [4].

Our previous studies suggested that TD in broiler chickens was in the normal process of proliferation of chondrocytes in the proliferative (PZ) and hypertrophic zones (HZ), but the TD causes death of chondrocytes, resulting in the destructed and mixed arrangements of chondrocytes in the PZ and HZ zones of growth plate correspondingly abnormally expressed in different genes within and above these chondrocyte lesions [1, 5, 6]. During long bone development, chondrocytes of the GP started proliferation. During proliferation, chondrocytes stack together to form a columnar layer of cells. Most distal cells of this columnar layer stop proliferation, leave the cell cycle, and differentiate into hypertrophic chondrocytes. These hypertrophic chondrocytes start mineralization of the surrounding matrix through the invasion of blood vessels. The apoptosis of hypertrophic chondrocytes allows blood vessel invasion, and finally, trabecular bone formation occurs by invading the cartilaginous matrix [7]. The cartilage cells of the GP go through proliferation, maturation, and hypertrophy and make matrix component X collagen [8]. TD considered causing the cartilage cell proliferation, nonvascularization, and nonmineralization of chicken GP [5, 6, 9].

Tetramethyl thiuram disulfide (thiram) is a pesticide, extensively used for experimental model due to its inducing effects like naturally occurring TD in broiler chicks [2, 4, 10, 11]. Previously, Tian et al. [11] have done a screening of differentially expressed genes (DEGs) in the GP of broiler chickens using thiram to induce TD in broiler chickens. The microarray analysis study found following histone deacetylase 1 (HDAC1), metastasis-associated 1 (MTA1), histone 4 (H4), and proliferating cell nuclear antigen (PCNA). These DEGs may be the cause of the proliferation of chondrocytes in TD chickens induced by thiram, which can be regulated by therapeutic intervention in the future.

Studies concerned with proliferation-related genes in the early stages of TD were not investigated previously. Therefore, this study has planned to investigate the proliferation-related genes HDAC1, MTA1, H4, and PCNA on RT-PCR and immunohistochemistry (IHC). Moreover, pathological changes of tibial GP compared to control and thiram-induced TD chickens were evaluated. This study may get insight into the pathogenicity of TD to find therapeutic intervention of TD.

## 2. Materials and Methods

### 2.1. Management, Treatment, and Sampling

360 Arbor Acres day-old chicks were purchased from Shanxi Daxiang Farming Group (Shanxi, China), and chicks were managed for 13 days. Feed was prepared following the guidelines of the National Research Council [12]. The basal diet and fresh water were provided on the basis of *ad libitum* basis during experimental trial. Before starting the experiments, chicks were acclimatized together for 7 days. At the end of 7^th^ day, chicks were fasted and divided into 8 groups. 45 chicks were allotted to each group with three replicates (15 chicks). The experiment was started on 8^th^ day (day 1). Thiram (JL131223002) was bought from American Research Product (Massachusetts, USA). In control groups without administration of thiram (days 1, 2, 4, and 6 groups), day 1 denotes the chicken of 8 days old, 2 denotes chicken of 9 days old, 4 denotes 11 days old, and 6 denotes 13-day-old chicken. The treatment groups were fed a diet containing 100 mg.kg^−1^ thiram for 48 hours (days 1, 2, 4, and 6 groups or T1, T2, T4, and T6). The chickens were anesthetized with ether and sacrificed for cervical dislocation in accordance with the reported method [13]. Finally, tibia samples were collected from each group (ten chicks).

### 2.2. Morphology and Histology

The cartilage growth plates were harvested from control and thiram-induced groups, photographed, then transferred into 4% paraformaldehyde, a part of frozen in liquid nitrogen, and stored at −70°C as described by Chen et al. [14]. The samples of right and left tibiotarsal bones were collected on days 1, 2, 4, and 6 from groups C and T for hematoxylin and eosin (HE) staining and immunohistochemistry (IHC). The right tibiotarsal bones were stored in 4% paraformaldehyde for HE and IHC, whereas left tibiotarsal bones were removed and photographed to analyze the morphology and the TD score as previously described by Rath et al. [15].

H&E staining was performed on the right GP, as previously described by Tian et al. [11]. Briefly, serial histological sections (4 *μ*m thickness) of the entire tibiae were prepared after samples had been fully decalcified in 10% ethylenediaminetetraacetic acid (EDTA). Decalcify fluid, dehydrate, embed in paraffin wax, and then stain with hematoxylin and eosin. The microscope examined sections, and prefixed samples were postfixed in 2% OsO4, dehydrated, and embedded in epoxy resin. Ultrathin sections were stained with uranyl acetate and lead citrate and observed with a HITACHI H-7650 transmission electron microscope at 80 kV and a Gatan 832 CCD camera.

Differential gene expression (HDAC1 and MTA1) was identified by IHC. The sections were deparaffinized in xylene, rehydrated through series of graded ethanol solutions, rinsed for 5 min in distilled water, and incubated in 3% H_2_O_2_ at room temperature for 10 min. HDAC1 and MTA1 were detected by IHC with HDAC1 and MTA1 antibodies at a 1 : 100 dilution using the Enhanced Sensitive IH Detection Kit I (BBCL, Wuhan, China) according to the manufacturer's protocol with rabbit anti-human HDAC1 polyclonal antibody (BA4267-2, Wuhan Boshide), rabbit anti-human MTA1 polyclonal antibody (BA4267-2, Wuhan Boshide), and SABC immunohistochemistry kit (rabbit IgG-1/2 KIT SA1022, Wuhan Boshide). As a negative control, nonimmune rabbit immunoglobulin G (IgG) was substituted for the primary antibody (Jahejo et al., 2020c). IHC profiler plugin of 1.48 version ImageJ software (NIH, Bethesda, Maryland) (Java 1.8.9) was used to determine the expression score of HDAC1 and MTA1 in control and thiram-treated groups, following the method described by Zhang et al. [16]. In this study, the method was determined by calculating the relative area of chondrocytes in percentage by dividing the area of interest of chondrocytes with the total area. Furthermore, the obtained data from IHC images were analyzed by independent sample *t*-test using SPSS 17.0 software as previously done in our research article [18].

### 2.3. RNA Extraction, PCR, and Real-Time qPCR

Total RNA samples were extracted from the tibial GP using TRIzol RNA purification kit (Takara, Japan). After extraction of total RNA samples from tibial GP tissue, the samples were used for PCR and RT-qPCR. Transcripts from each sample were amplified in triplicate and detected using an SYBR Green PCR Master Mix (Applied Biosystems). Premier 5.0 software was used to design PCR primers based on the sequences of the HDCA1, MTA1, H4, and PCNA and housekeeping 18s rRNA reference gene in the NCBI GenBank. The primer sequences are shown in Table 1. The primers were synthesized by shanghai Generay Biotech Co.,Ltd. Operate according to the instruction of SYBR® Premix Ex Taq II (Takara, Dalian), and use QuantStudio 6 system (ABI, USA) for PCR reaction.

## 3. Results

### 3.1. Morphology and Pathology of Chicken Tibial Growth Plate

**Figure 1:**
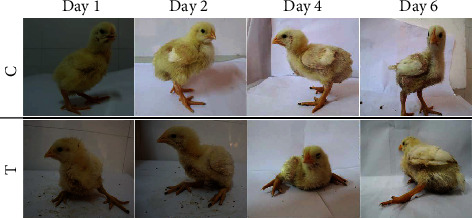
Clinical observation of lameness in control vs. thiram- induced tibial dyschondroplasia (TD) in chicks on days 1, 2, 4, and 6.

**Figure 2:**
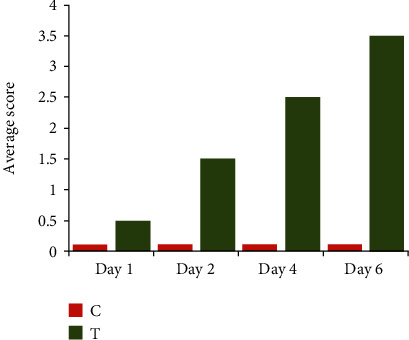
Comparison of average TD lesion score in control vs. thiram-induced TD in chicks on days 1, 2, 4, and 6. C = control; T = thiram. Following Pines et al. [31], the average TD score was calculated.

**Figure 3:**
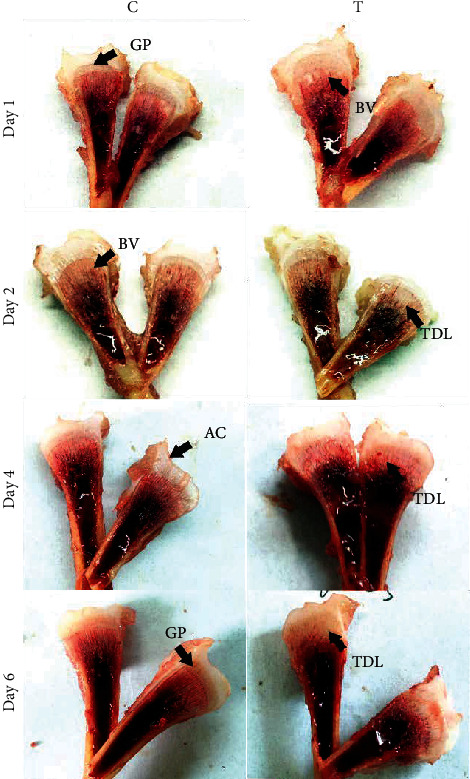
Comparison of the growth plate in control vs. and thiram-induced TD broiler chickens on days 1, 2, 4, and 6. C = control; T = thiram; GP = growth plate; BV = blood vessels; TDL = tibial dyschondroplasia lesions; AC = articular cartilage.

The clinical observation showed that the broiler chicks of control groups were active and healthy, while thiram-treated groups (days 1, 2, 4, and 6) chicks were moving with difficulty along with lameness. The lameness was observed less to worse with the passage of time (days 1 to 6) progressed (Figure 1). TD score was compared between control vs. thiram-induced chicks, and TD score was high on day 6 while low on day 1 (Figure 2). The tibial growth palate was smooth and well developed in control chicks, while opaque rigid mass in thiram-induced chickens on days 1-6 thickened insistently with progress in days (Figure 3).

### 3.2. Histology of Chicken Tibial Growth Plate

**Figure 4:**
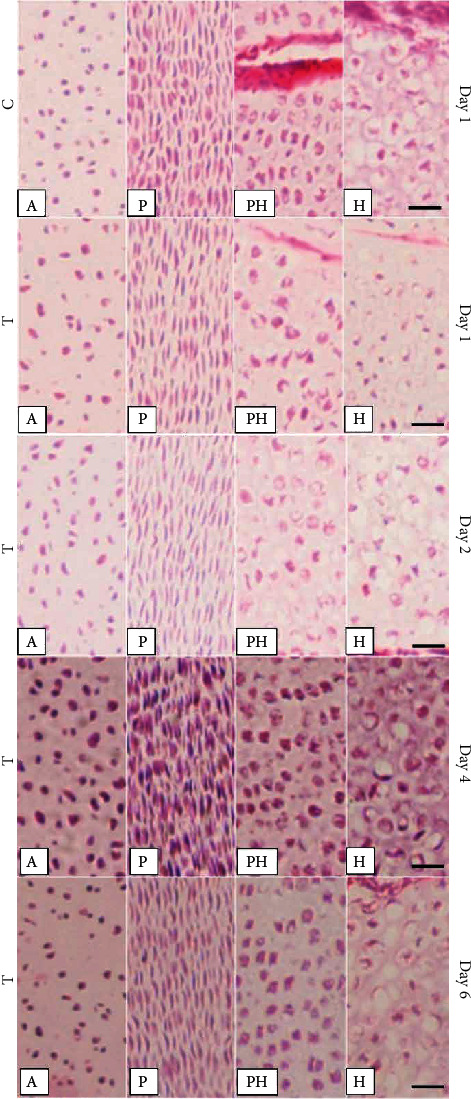
The histological characteristics of chondrocytes in the different zones of the tibia growth plate on days 1, 2, 4, and 6 in control vs. thiram-induced TD. C = control; T = thiram; A = articular zone; P = proliferative zone; PH = prehypertrophic zone; H = hypertrophic zone (bar = 50 *μ*m).

The histological H&E staining results of groups C and T shared a similar pattern as pathological results. The arrangement of chondrocytes degenerated, damaged, and diminished in group T for 1, 2, 4, and 6 days. However, it can be observe that less to more degeneration and damage of thiram-induced TD chicken chondrocytes were found from day 1 to 6 in the proliferative zone (PZ), prehypertrophic zone (PH), and hypertrophic zone (HZ) (Figure 4). The chondrocytes were partially rounded at the PZ on day 1 in group T. Moreover, the proliferative chondrocytes were also distributed in the PH and HZ. Tibial GP on day 4 in group T showed proliferation, and chondrocytes accumulated in PH and HZ. The number of chondrocytes decreased further and appeared as pyknotic cells. Most importantly, the chondrocytes on day 6 in group T for PZ and HZ densely accumulated in the PH, which might result atrophy of blood vessels and angionecrosisformed a dense, rigid avascular mass at the tibial GP.

### 3.3. Agarose Gel Electrophoresis

**Figure 5:**
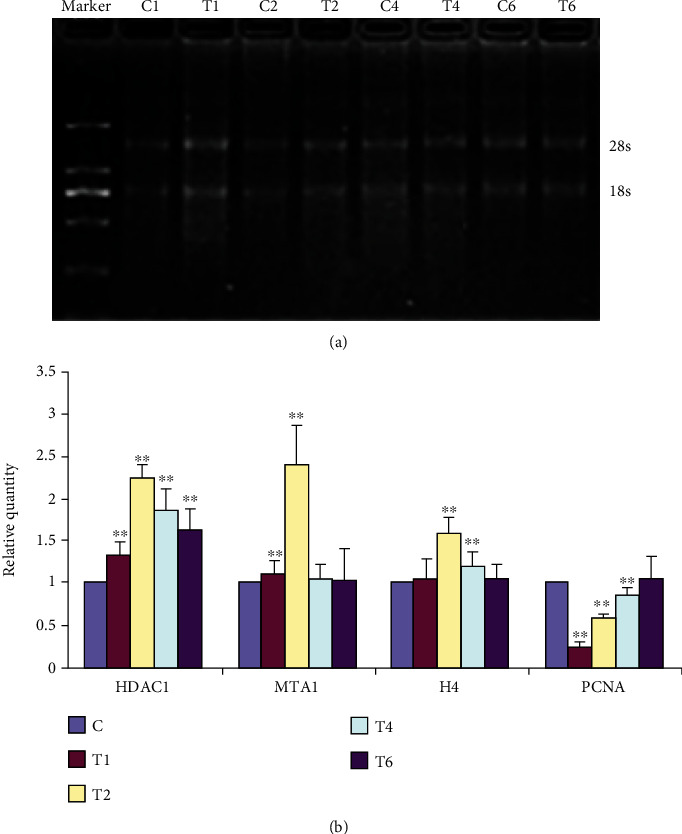
Analysis of gene expression. (a). Analysis of RNA detected by agarose gel electrophoresis. (b). Gene expression analysis by qRT-PCR. C = control; C1 = control day 1; T1 = thiram day 1; C2 = control day 2; T2 = thiram day 2; C4 = control day 4; T4 = thiram day 4; C4 = control day 4; T4 = thiram day 4. ∗ indicates significant differences (*P* < 0.05).

The role of proliferative-related genes HDAC1, MTA1, H4, and PCNA was evaluated in thiram-induced TD chickens at an early stage. Prior to qRT-PCR, agarose gel electrophoresis was performed, which shows clear bands of 28 s and 18 s. The brightness ratio was close to 2 : 1. The microultraviolet spectrophotometer measures the quality of each group of RNA samples. The ratio of A260/A280 is between 1.9 and 2.1, and the ratio of A260/A230 is above 2.0, indicating that the RNA samples are not contaminated (Figure 5(a)).

### 3.4. mRNA Expression of Proliferative-Related Genes

To reveal the role of proliferative-related genes (HDAC1, MTA1, H4, and PCNA), expressions were detected by real-time PCR. The results showed that the mRNA transcription levels of HDAC1 gene in the tibial GP of thiram-induced TD chicken group T was significantly (*P* < 0.01) upregulated on days 1, 2, 4, and 6 as compared to group C. The mRNA expression of MTA1 and H4 gene was upregulated in group T, among which the difference was observed highly significant at day 2 (*P* < 0.01). The mRNA expression level of the PCNA gene was significantly (*P* < 0.01) downregulated at days 1, 2, and 4 in group T (Figure 5(b)).

### 3.5. Immunohistochemistry Analysis

**Figure 6:**
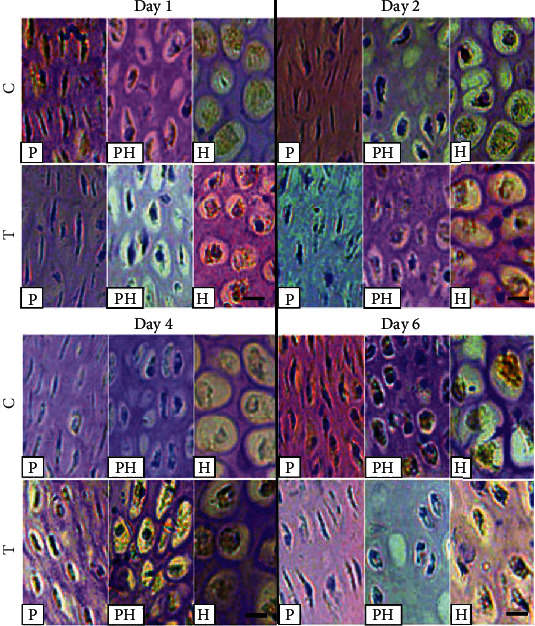
The immunohistochemistry analysis of HDAC1 protein expression in the tibial growth plate of different chondrocytes zones on days 1, 2, 4, and 6 in control and thiram-induced tibial dyschondroplasia. C = control; T = thiram; A = articular zone; P = proliferative zone; PH = prehypertrophic zone; H = hypertrophic zone (bar = 50 *μ*m).

Based on the mRNA expression of RT-qPCR, two highly expressed genes HDAC1 and MTA1 were selected to evaluate their protein expression changes in the chondrocytes using IHC. This study quantified the localization of HDAC1 protein expression using IHC, in the chondrocytes of thiram-induced TD chicken group T as compared to group C (Figure 6 and Table 2). The results suggest that HDAC1 was significantly (*P* < 0.01) expressed in the PZ and HZ of group T on days 1 and 2. HDAC1 protein on day 4 was significantly (*P* < 0.01) expressed in the chondrocytes of group T for PZ, PHZ, and HZ. However, the expression of HDAC1 was significantly (*P* < 0.01) expressed for day 6 in the HZ.

**Figure 7:**
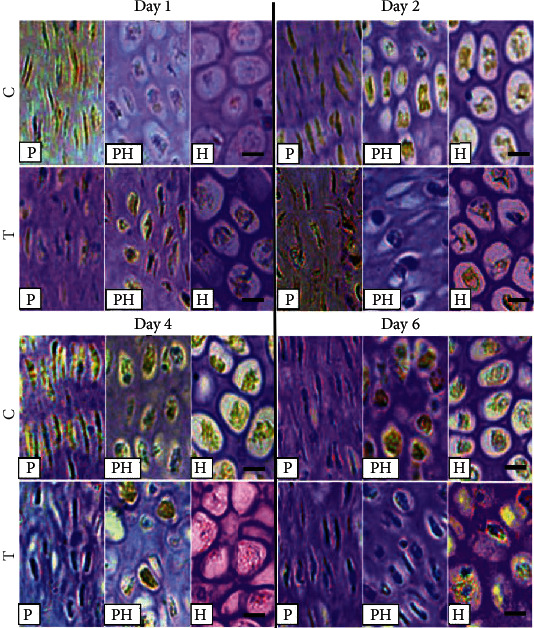
The immunohistochemistry analysis of MTA1 protein expression in the tibial growth plate of different chondrocytes zones on days 1, 2, 4, and 6 in control and thiram-induced tibial dyschondroplasia. C = control; T = thiram; A = articular zone; P = proliferative zone; PH = prehypertrophic zone; H = hypertrophic zone (bar = 400 *μ*m).

This study quantified the localization of MTA1 protein expression using IHC, in the chondrocytes of thiram-induced TD chicken group T as compared to group C (Figure 7 and Table 2). The expression level of MTA1 in the thiram-induced TD chicken tibial GP was similar to HDAC1 expression results. MTA1 expression significantly increased (*P* < 0.01) in the thiram-induced TD chicken group T as compared to group C on day 1. MTA1 expression in the chondrocytes of thiram-induced TD chicken group T significantly upregulated (*P* < 0.01) in PZ and HZ on day 2. On the 4th and 6th days of the T group, the expression of MTA1 was significantly upregulated in PZ, PHZ, and HZ (*P* < 0.01).

## 4. Discussion

The pathogenicity related to TD is still not well established completely. The reported studies conducted so far presented many possible pathogenicity preventive measures [17–22], but the problem has not been completely evacuated yet. Therefore, any related study which can add into the possible pathogenicity of TD is valuable. Proliferation of chondrocytes is very important for the tibial GP, and the role of proliferation-related genes in thiram-induced TD chickens has not been studied yet. This study planned to find the role of proliferation-related genes in thiram-induced TD chickens, and the genes were selected based on our previous microarray study [11].

The clinical results of thiram-induced TD chicken groups observed that the lameness was observed more on day 6 in group T. Moreover, the histopathological results of this study suggested that damaged chondrocytes were seen higher on day 6 as compared to days 1, 2, and 4. The changings in the chondrocytes arrangements of the thiram-induced TD chicken could be due to the abnormal proliferation and apoptosis. The chondrocytes stopped growing and multiplying, and ultimately, hypertrophy and apoptosis in tibia occurred [4, 5, 13, 17]. The chondrocytes of the GP normally arranged in columns and could not easily be distinguished due to high density and less extracellular matrix in TD [15, 23]. Thiram could promote chicken GP chondrocyte proliferation and disturb the regulation of endochondral calcification and the development of normal cartilage. In addition, it may result in prehypertrophic cell accumulation, angionecrosis, abnormal extracellular matrix synthesis, defer endochondral calcification, and bone resorption. In this study, we found that the number, morphology, arrangement, and distribution of chondrocytes have been changed. Thiram destroyed the regular columns of chondrocytes, and the number of cells decreased in the tibial GP of broiler chickens [16, 24, 25].

The mRNA expression levels of HDAC1 in thiram-induced TD chicken groups were analyzed. The results of this experiment were found consistent with the study on the relationship between HDAC1 and cancer, which was observed high in abnormal proliferation [26, 27]. The mRNA expression of histone H4 gene was upregulated on days 1 and 2 in thiram-induced TD chickens. It could be related to the proliferation of chondrocytes in the GP, which intensifies DNA replication and accumulates simultaneously. However, the mRNA expression level of histone H4 gene returned to normal on day 6 in group T, which could be related to other ways of regulating the TD injury that continues to intensify after feeding thiram. The mRNA expression level of MTA1 gene significantly changed in thiram-induced TD chicken group T. The pathological results of this study showed that the TD injury developed for a long time after feeding thiram, and the cell proliferation did not show signs of weakening, indicating that the change of MTA1 gene mRNA level was only related to feeding thiram. MTA1 protein can be expressed in various zones of tibial GP, and the results were consistent with the tumor [28–30]. This shows that the differential expression of MTA1 protein is related to the time of thiram feeding. The expression of PCNA gene mRNA expression was downregulated on days 1 and 2 after feeding thiram as compared to the control group. The reason for this result could be due to the GP chondrocytes, which proliferate differently than the tumor cells. The mRNA expression of the PCNA gene increased throughout the experimental period. It could be due to the rapid decrease of cell proliferation.

## 5. Conclusion

It was concluded that proliferation-related genes HDAC1, MTA1, H4, and PCNA were differentially expressed in the tibial GP of thiram-induced TD chickens. Moreover, HDAC1 and MTA1 protein could be responsible for the abnormal proliferation of chondrocytes in the tibial GP of thiram-induced TD chicken group. This research may provide new insights into the therapeutic targets of these proliferation-related biomarker genes to eradicate TD at an early stage.

## Figures and Tables

**Table 1:** Primer sequences for qRT-PCR.

Genes	Primer sequences	Accession no.	Product size (bp)
18S rRNA	F: TTCCGATAACGAACGACAC	FM165414	139
	R: GACATCTAAGGGCATCACAG		
HDAC1	F: GAGACTGCTGTGGCTTTG	NM_204156	104
	R: ATTGAGGGACTGATGTGC		
MTA1	F: GGAGAGAGACCAGGACCAAA	NM_001012953	171
	R: ATGCCAGGGGCGTAAGAT		
H4	F: GCGACAACATCCAGGGCAT	NM_001037843	155
	R: ATCGGTGTAGGTGACGGCG		
PCNA	F: AAGCACCAAATCAGGAAAAG	NM_204170	179
	R: GGCACAGGAGATGACAACA		

F = forward primer; R = reverse primers; bp = base pairs.

**Table 2:** The quantitative expression of HDAC1 and MTA1 protein. IHC profiler plugin of 1.48 version ImageJ software (NIH, Bethesda, Maryland) (Java 1.8.9) was used to determine the expression score of HDAC1 and MTA1 in control and thiram-induced TD chicken groups, following the method described by Zhang et al. [16].

	Groups	PZ	PHZ	HZ
HDAC1	Day 1-C	138.299 ± 4.466	150.736 ± 3.832	150.804 ± 4.550
	Day 1-T	127.539 ± 2.281^∗∗^	147.594 ± 4.561	145.596 ± 2.962^∗^
	Day 2-C	111.285 ± 2.852	113.556 ± 1.963	124.801 ± 1.807
	Day 2-T	106.846 ± 1.935^∗^	118.172 ± 0.858	119.593 ± 144.2^∗^
	Day 4-C	132.482 ± 1.350	132.345 ± 4.123	125.459 ± 3.209
	Day 4-T	83.345 ± 2.408^∗∗^	82.231 ± 1.527^∗∗^	93.237 ± 3.431^∗∗^
	Day 6-C	112.191 ± 1.650	113.506 ± 1.621	113.072 ± 1.954
	Day 6-T	111.150 ± 1.762	111.475 ± 1.943	123.941 ± 3.400^∗∗^

MTA1	Day 1-C	163.235 ± 4.537	163.505 ± 3.857	160.401 ± 1.449
	Day 1-T	113.150 ± 3.655^∗∗^	115.208 ± 2.079^∗∗^	113.164 ± 1.441^∗∗^
	Day 2-C	140.132 ± 4.543	139.559 ± 2.165	140.223 ± 2.906
	Day 2-T	125.932 ± 5.002^∗∗^	138.727 ± 3.106	130.010 ± 3.112^∗∗^
	Day 4-C	135.383 ± 1.780	130.626 ± 2.151	127.679 ± 2.309
	Day 4-T	80.582 ± 3.508^∗∗^	81.261 ± 1.527^∗∗^	91.847 ± 1.731^∗∗^
	Day 6-C	128.790 ± 1.792	133.276 ± 1.330	133.101 ± 2.634
	Day 6-T	116.187 ± 1.908^∗∗^	119.894 ± 2.384^∗∗^	122.536 ± 2.613^∗∗^

C = control; T = thiram; PZ = proliferative zone; PHZ = prehypertrophic zone; HZ = hypertrophic zone. ∗ indicates significant differences (*P* < 0.05).

## Data Availability

No data were used to support this study.
